# Association between waist-to-hip ratio and risk of myocardial infarction: a systematic evaluation and meta-analysis

**DOI:** 10.3389/fcvm.2024.1438817

**Published:** 2024-12-09

**Authors:** Xiaojuan Zhang, Liu Yang, Cong Xiao, Jiacong Li, Tao Hu, Linfeng Li

**Affiliations:** ^1^ Medical College of Nanchang University, Jiangxi Provincial People’s Hospital, Nanchang, Jiangxi, China; ^2^ Department of Cardiology, Jiangxi Provincial People’s Hospital, The First Affiliated Hospital of Nanchang Medical College, Nanchang, Jiangxi, China

**Keywords:** waist-to-hip ratio, myocardial infarction, central obesity, meta-analysis, incidence rate

## Abstract

**Background:**

Myocardial infarction(MI) is one of the most serious health threats. Despite the increasing number of clinical methods used to predict the onset of MI, the prediction of MI is still unsatisfactory and necessitates new methods.

**Objective:**

To systematically review observational studies from the past two decades on the association between waist-to-hip ratio (WHR) and MI risk.

**Methods:**

Original literature on the correlation between WHR and MI was searched in PubMed, Embase, Web of Science, Cochrane Library, Science Direct, CNKI, and Wanfang up to January 31, 2024. Two researchers independently screened, extracted data, and assessed quality using the Newcastle-Ottawa Scale (NOS) and Revman5.3. Meta-analysis with Stata 16.0 calculated the combined Odd ratio (OR) for WHR and MI risk. Heterogeneity was assessed with the *I*^2^ statistic to select the appropriate effects model. Subgroup analysis, meta-regression, sensitivity analysis, and funnel plots tested for heterogeneity and publication bias.

**Results:**

A total of 22 observational studies were included, involving 709,093 participants. The meta-analysis showed that an elevated WHR was significantly associated with an increased risk of MI, with a pooled odds ratio (OR) of 1.98 [95% Confidence interval (CI): 1.75–2.24] and high heterogeneity (*I*^2^ = 91.5%, *P* < 0.0001). Subgroup analysis revealed a stronger association between WHR and MI in women (OR: 1.99, 95% CI: 1.43–2.77) compared to men (OR: 1.74, 95% CI: 1.36–2.22). Regional analysis indicated that the association between WHR and MI risk was highest in Asian populations (OR: 2.93 95% CI: 1.61–5.33), followed by American (OR: 1.73, 95% CI: 1.45–2.08) and European populations (OR: 2.19, 95% CI: 1.49–3.22). Sensitivity analysis demonstrated that the results remained stable after excluding one study.

**Conclusion:**

In the general adult population, a higher WHR is a potentially significant association for MI and has predictive value for MI.

## 1 Background

MI is a serious cardiovascular disease, with symptoms including severe chest pain, tightness, and difficulty breathing (1, 2). If not treated promptly, it often leads to serious complications or even death (2). Therefore, identifying valuable risk factors to help predict MI would promote healthcare. There is a large body of research indicating that obesity-related cardiometabolic diseases are risk factors for atherosclerotic cardiovascular disease (3). Per the WHO and numerous other internationally recognized organizations like the CDC, obesity is defned by body mass index(BMI), an indirect measure of body composition. However, patients can be at an increased risk of cardiometabolic diseases if they have a normal BMI but an elevated body fat percentage (i.e., “normal weight obesity”) (4). It is therefore important to consider other measures of body composition besides BMI as a measure of body fat and predict cardiometabolic risk. Dual-energy x-ray absorptiometry (DEXA) (5), bioimpedence analysis (BIA) (5), computed tomography (CT) and magnetic resonance imaging (MRI) are direct measures of body fat,while WHR, waist-to-height ratio (WHtR) and waist circumference (WC) are other indirect measures of body fat besides BMI. DEXA is a technique for measuring bone density and body fat content using the principle that different energy x-rays are absorbed to different degrees in human tissues (5). BIA is used to predict body composition based, on the electrical conductive properties of the body (5). Among these, DEXA, CT, and MRI are expensive and not readily available.BMI cannot distinguish between local and peripheral fat and does not accurately reflect the impact of WC and height (6, 7). Additionally,WC has been found in some studies to not predict the prognosis of MI well (7).

The WHR is an indicator of central obesity to predict the incidence and prognosis of cardiovascular disease. Overall, WHR as an indicator of central obesity is superior to other indicators. It not only predicts the incidence of MI but also has reference value for predicting myocardial injury before MI (8), the prognosis of MI (9, 10), and the severity of MI in patients (11, 12). The WHR is usually used as an indicator of central obesity to predict the incidence and prognosis of cardiovascular diseases. The waist circumference divided by the hip circumference defines the WHR, and the World Health Organization recommends a WHR ≥ 0.9 for men and ≥ 0.85 for women as the standard diagnostic criteria for abdominal obesity (13).

To clarify the association between WHR and MI, this paper reviews the research on the association between WHR and the risk of MI over the past two decades, summarizes the results of these studies in a meta-analysis, and aims to elucidate the relationship between WHR and MI in the general adult population. In particular, this study adds the latest data to previous studies (14, 15) and conducts a more detailed subgroup analysis, which further enriches the existing literature, especially in terms of gender, regional differences, and long-term risk assessment.

## 2 Materials and method

### 2.1 Search strategy

Computerized searches were conducted in databases such as PubMed, Embase, Web of Science, Cochrane Library, Science Direct, CNKI, and Wanfang. The English search terms included: Ratio, Waist-Hip; Ratios, Waist-Hip; Waist Hip Ratio; Waist-Hip Ratios; Waist-to-Hip Ratio; Ratio, Waist-to-Hip; Ratios, Waist-to-Hip; Waist to Hip Ratio; Waist-to-Hip Ratios; Myocardial Infarction; Cardiovascular Stroke; Cardiovascular Strokes; Stroke, Cardiovascular; Strokes, Cardiovascular; Myocardial Infarct; Myocardial Infarcts; Heart Attack; Heart Attacks. The Chinese search terms included: myocardial infarction, acute myocardial infarction, inferior wall myocardial infarction, anterior wall myocardial infarction, anteroseptal myocardial infarction, ST-segment elevation myocardial infarction, non-ST-segment elevation myocardial infarction, coronary atherosclerotic heart disease, angina, acute coronary syndrome. Two scholars from the team independently searched the above databases, with the search time up to January 31, 2024.10.21.

### 2.2 Inclusion and exclusion criteria for the literature

Criteria for Literature Inclusion: (1) The study subjects were the general population aged over 18 years; (2) The studies were reported in Chinese or English; (3) The studies explored the correlation between WHR and the incidence rate of MI; (4) The studies adjusted for various potential influencing factors on the association between WHR and the incidence rate of MI.

The exclusion criteria were as follows: (1) Non-clinical human trials; (2) Studies with questionable data or data that could not be extracted; (3) Literature that did not explore the WHR and the incidence rate of MI.

### 2.3 Literature screening

According to the specified time limit (up to January 31, 2024), two scholars independently screened the retrieved original studies. First, using Endnote to compare the titles, publication years, first authors' names, etc., to exclude duplicate literature. Then, by reading the titles and abstracts, literature unrelated to the research purpose was eliminated. Next, the remaining literature was fully searched and read, and the original studies to be finally included were confirmed according to the inclusion and exclusion criteria. In case of disagreement between the two researchers, a third researcher was involved for verification and assessment.

### 2.4 Data extraction

Two researchers read the papers and extracted relevant data, including the main authors' surnames, publication years, study regions, methods, sample sizes, subjects' ages, WHR, types of MI, WHR cut-off values, OR/RR/HR (95%CI), gender grouping, and adjustment factors in multivariate analysis.

### 2.5 Quality assessment

The final included studies covered case-control studies and cohort studies. Two methods were used for the quality assessment of the literature: firstly, the NOS was used to assess multiple aspects of case-control studies and cohort studies, mainly including the representativeness of the studies, comparability between groups, and measurement of exposure factors, with a total score of 9 points, and studies scoring above 6 points were considered high-quality research. Subsequently, Revman5.3 software was used for assessment, focusing on random sequence generation, allocation concealment, blinding, outcome assessment blinding, completeness of outcome data, selective reporting, and other potential biases.

### 2.6 Data analysis

This study utilized Stata 16.0 software to perform meta-analysis and statistical analysis. The analyzed data were categorical variables, presented as OR and 95% CI to demonstrate the association between WHR and MI in the general adult population. Heterogeneity among studies was assessed using Cochrane's *Q* test and the *I*^2^ statistic. A fixed-effect model was adopted if *I*^2^ < 50%; a random-effect model was used if *I*^2^ > 50%. Significant heterogeneity was considered present if the *P*-value of the *Q* test was <0.05 and *I*^2^ > 50%, in which case subgroup analysis and multivariate meta-regression analysis were conducted to explore the sources of heterogeneity. Subgroup analyses included gender (male, female), study region (Asia, Europe, America), type of study (case-control study, cohort study), and WHR cut-off value (<0.93, ≥0.93). The chi-square test was used to compare results within subgroups. If one subgroup had *I*^2^ < 50% and *P* > 0.05, while another had *I*^2^ > 50% and *P* < 0.05, it indicated that this subgroup might be the source of heterogeneity. Multivariate meta-regression analysis was also used, incorporating factors such as NOS score, average age, publication year, etc., that might affect the results into the model to investigate their potential impact on the study outcomes. If *P* < 0.05, it suggested that these factors might be sources of heterogeneity. Sensitivity analysis was conducted by excluding studies one by one to verify the stability of the results. If the OR values were stably distributed on both sides of the median line, it indicated that the results of the meta-analysis were stable. Potential publication bias was checked by visually inspecting the symmetry of the funnel plot. If the funnel plot was symmetrical, it suggested a lower risk of publication bias; if the results clustered on one side of the plot, it might indicate the presence of publication bias.

## 3 Results

### 3.1 Literature search results

**Figure 1:**
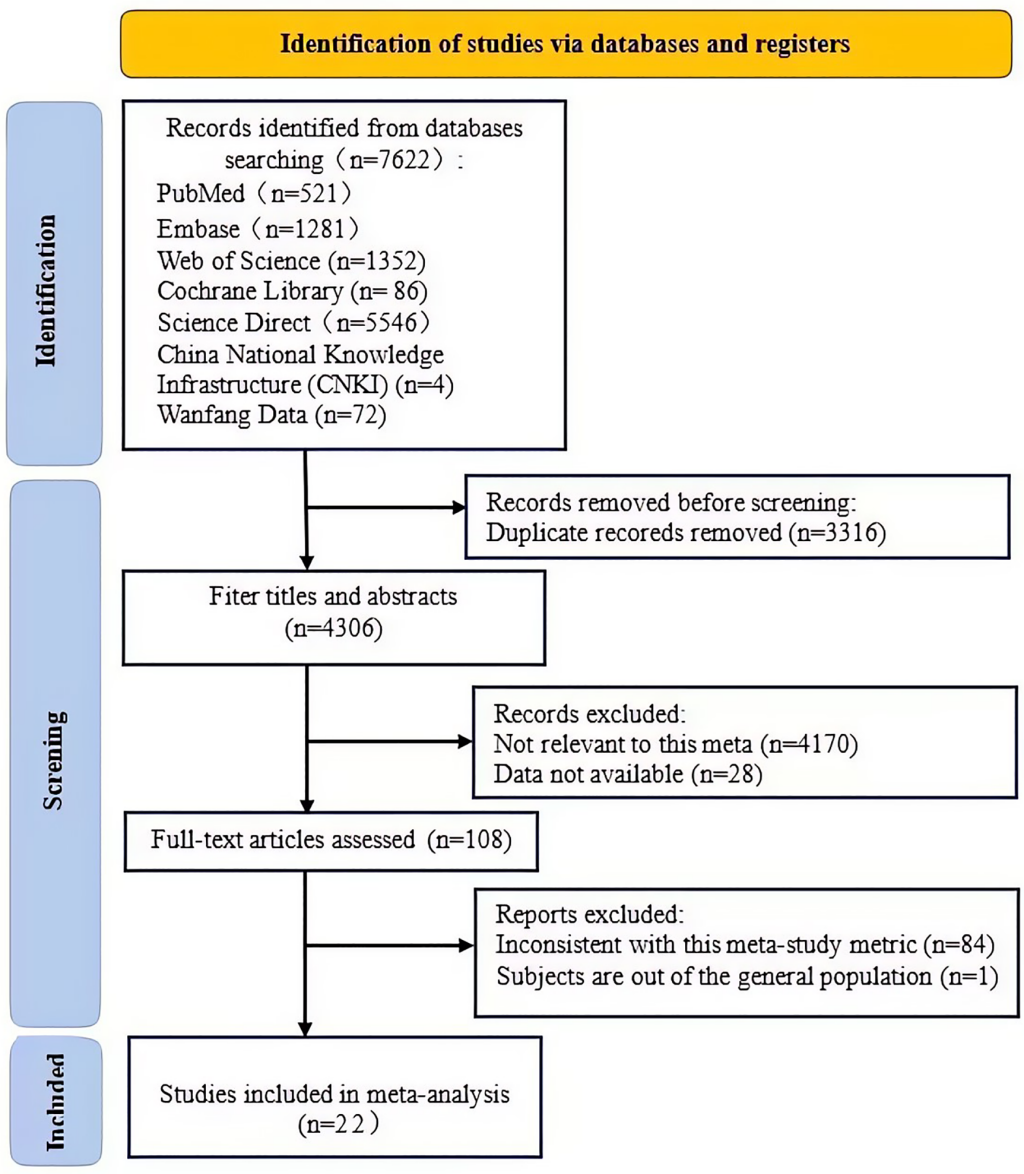
Flow diagram of search and study selection.

**Figure 2:**
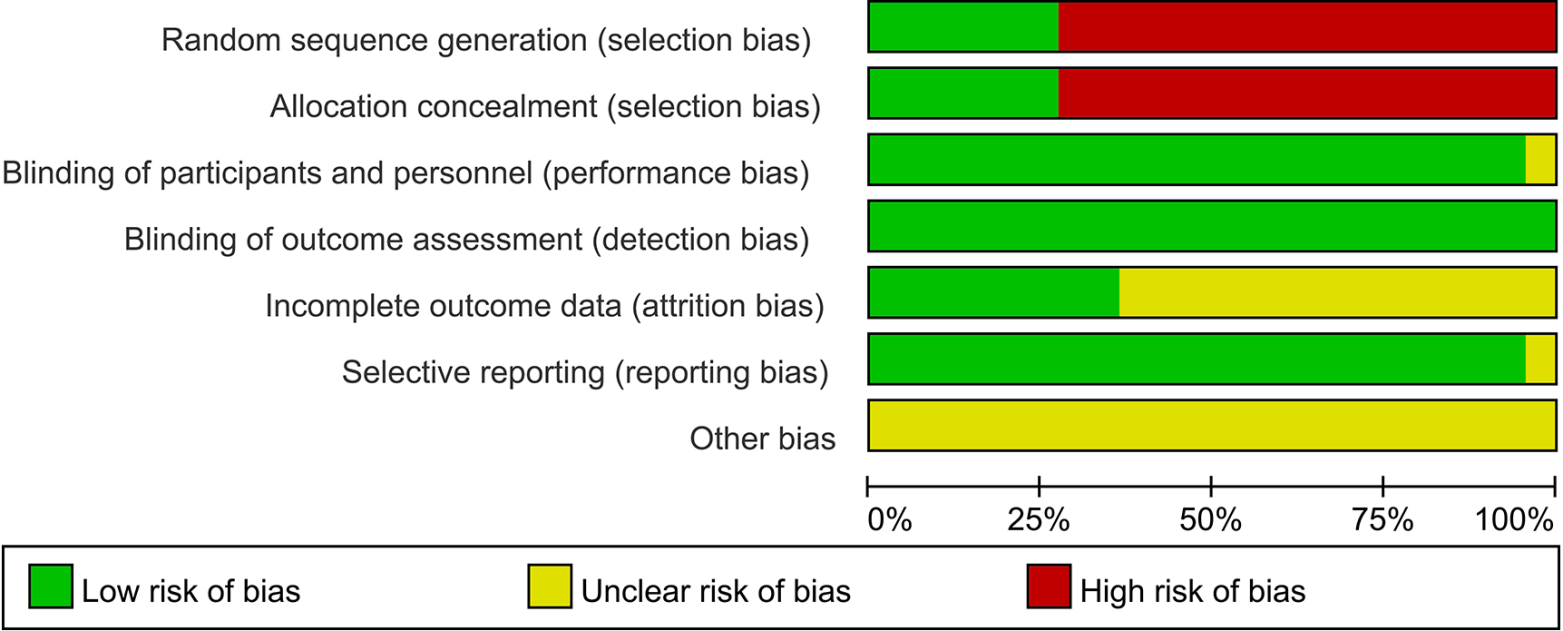
Quality assessment 1.

We searched for English keywords in databases such as PubMed, Embase, Web of Science, Cochrane Library, Science Direct, and for Chinese keywords in CNKI and Wanfang Database, retrieving a total of 7,622 related articles. Among them, there were 521 from PubMed, 1,281 from Embase, 1,352 from Web of Science, 86 from Cochrane Library, 5,546 from Science Direct, 4 from CNKI, and 72 from Wanfang Database. After selection using Endnote and removing 3,316 duplicate articles, 4,306 remained. By reviewing titles and abstracts, and applying inclusion and exclusion criteria, 4,198 articles were screened out, including 4,170 unrelated to the study (not discussing the association between WHR and MI) and 28 unable to obtain complete data (no online access to full text, incomplete data, or obviously abnormal data). After full-text reading of the remaining 108 articles and another round of screening with inclusion and exclusion criteria, 85 articles were excluded, among which 84 had inconsistent research indicators (did not report the OR/HR/RR values of WHR and MI risk), and 1 involved a non-general population. Finally, 22 articles met the criteria (16–37). For the specific screening process, please refer to Figures 1, 2.

### 3.2 Literature inclusion and quality assessment

This study included 22 observational studies, comprising 7 cohort studies and 15 case-control studies, spanning from 1996 to 2023. These studies encompassed multiple countries and regions, such as Europe, Asia, and South America, with a total of 709,093 participants. The age range of the subjects was from 31.1 to 69.7 years, with WHR cut-off values ranging from 0.78 to 0.95, and the OR values indicating the association between an increase in WHR and the risk of MI ranged from 1.049 to 10.9. Among these, 8 studies also included gender subgroup analyses. When exploring the association between WHR and MI, studies typically adjusted for various factors, such as age, gender, body mass index, smoking, drinking, systolic blood pressure, total cholesterol, high-density lipoprotein, triglycerides, glycated hemoglobin, and other potential confounding factors, please refer to Table 1. The included studies encompassed seven cohort studies, which furnished data regarding the incidence of MI, methods of follow-up, and duration of follow-up. Please refer to Table 2.

**Table 1:** Specific characteristics of the included studies2.

Study	Gender	WHR cutt-of value	OR or RR or HR (95%CI)	Variable adjustment	NOS
Pais P^16^	Total	0.92	3.12 (1.80–5.40)	Smoking, hypertension, income, non-vegetarianism and fasting blood sugar	7
Hertzel C^17^	Total	0.89	2.41 (1.75–3.31)	Age and gender	8
Azevedo A^18^	Male	–	2.50 (1.30–4.90)	Age, education level, family history of acute myocardial infarction and smoking	8
	Female	–	3.00 (0.60–14.60)		
Leopoldo S^19^	Total	–	1.52 (1.06–2.18)	Smoking, blood sugar, family history of coronary heart disease, low-density lipoprotein-cholesterol, hypertension, diabetes and drinking	8
Kumar P^20^	Total	–	5.20 (1.40–21.10)	History of acute myocardial infarction, smoking, BMI, hypertension, total cholesterol, serum triglyceride, LDL, HDL, blood lipid and history of apolipoprotein E4 genotyping	9
	Male	0.95			
	Female	0.80			
Yusuf S^21^	Total	–	1.77 (1.67–1.88)	Age, gender, regiom and smoking	8
	Male	0.90			
Avezum A^22^	Female	0.83			
	Total	–	3.07 (1.66–5.66)	Age and gender	7
Lanas F^23^	Total	–	2.49 (1.97–3.14)	Age, gender and smoking	7
	Male	0.90			
	Female	0.83			
Kumar A^24^	Total	0.95	3.90 (2.10–6.30)	Age, gender and hospital	8
Oliveira A^25^	Total	–		Age, educational attainment, drinking, smoking, physical activity, family history of cerebral infarction and the impact of menopause and hormone replacement therapy on women	8
	Male	0.90	10.90 (6.10–19.4)		
	Female	0.85	5.84 (3.37–10.10)		
Carević V^26^	Total	–	1.96 (1.21–3.18)	Age and gender	7
	Male	0.90			
	Female	0.83			
Kaur R^27^	Total	–	4.80 (3.20–7.30)	Confounding effects of traditional coronary risk factors	9
	Male	0.80			
	Female	0.95			
Horvei LD^28^	Male	0.95	2.50 (1.30–4.90)	Age, smoking, systolic blood pressure, total cholestrol, density lipoprotein, triglyceride, glycated hemoglobin and diabetes	8
	Female	0.85	1.09 (0.60–14.60)		
Egeland GM^29^	Male	0.91	1.22 (1.07–1.40)	Age, smoking, bmi, systolic blood pressure, diabetes and total cholesterol-hdl cholesterol ratio	9
	Male	0.91	1.09 (0.97–1.23)		
	Female	0.80	1.76 (1.37–2.25)		
	Female	0.80	1.05 (0.90–1.24）		
Rådholm K^30^	Total	–	1.08 (1.00–1.18)	Age, gender, smoking, region and randomized antihypertensive and hypoglycemic interventions	8
Peters SAE^31^	Male	–	1.36 (1.30–1.43)	Age, townshend deprivation index and smoking	9
	Female	–	1.40 (1.39–1.59）		
Hermansson J^32^	Male	1.00	1.47 (0.97–2.24)	Age and work system	7
	Female	0.88	4.17 (2.19–7.92)		
Calling S^33^	Total	0.78	1.80 (1.34–2.42)	Postmenopausal treatment,age at menopause, drinking and family history of cardiovascular disease	9
Upadhyay R^34^	Total	–	1.74 (1.02–2.94）	Age, gender and types of residential areas	8
	Male	0.95			
	Female	0.85			
Li Y^35^	Total	–	1.34 (0.46–3.85)	Gender, age, bmi, diabetes, drinking, fasting blood sugar, heart rate, hdl, hypertension, ldl, physical activity, salt consumption, systolic blood pressure and smoking	9
	Male	0.92			
	Female	0.89			
Wienbergen H^36^	–	0.87	1.57 (0.82–2.99)	Age, gender, nation, level of education, smoking, drinking, bmi, hypertension and diabetes	8
	–	0.93	6.27 (3.40–11.54)		
Zhong P^37^	Total	–	1.43 (1.15–1.78)	Age, gender, racist, income, level of education, lifestyle and history of current drug use	9
	Male	0.90			
	Female	0.85			

**Table 2:** Specific characteristics of the Cohort studies.

Study	Incidence of MI	Gender and year	For follow-up methods	Follow-up time(year)
Horvei LD^28^	1.13%	–	Access to medical records	15
Egeland GM^29^	2.90%	Male, year < 60	Every Norwegian resident has a unique personal identification number, which is used to identify individuals through linkage with records from the Norwegian Cause of Death Registry and the National Hospital Discharge Diagnosis Data. In clinical drug trial studies	7
	11.80%	Male, year ≥ 60		
	0.70%	Female, year < 60		
	7.40%	Female, year ≥ 60		
Rådholm K^30^	7.00%	–	Follow-up includes regular blood draws, among other procedures	9
Peters SAE^31^	1.20%		In the UK Biobank cohort study, follow-up includes regular blood draws, among other procedures.	7
Calling S33	3.10%	–	Access to medical records	17
Li Y^35^	0.46%	–	In the Kailuan prospective cohort study, questionnaires and laboratory tests are repeated every two years as part of the follow-up process.	6
Zhong P^37^	3.70%	–	In the UK Biobank cohort study, participants are followed up with regular blood draws and other procedures.	12

**Figure 3:**
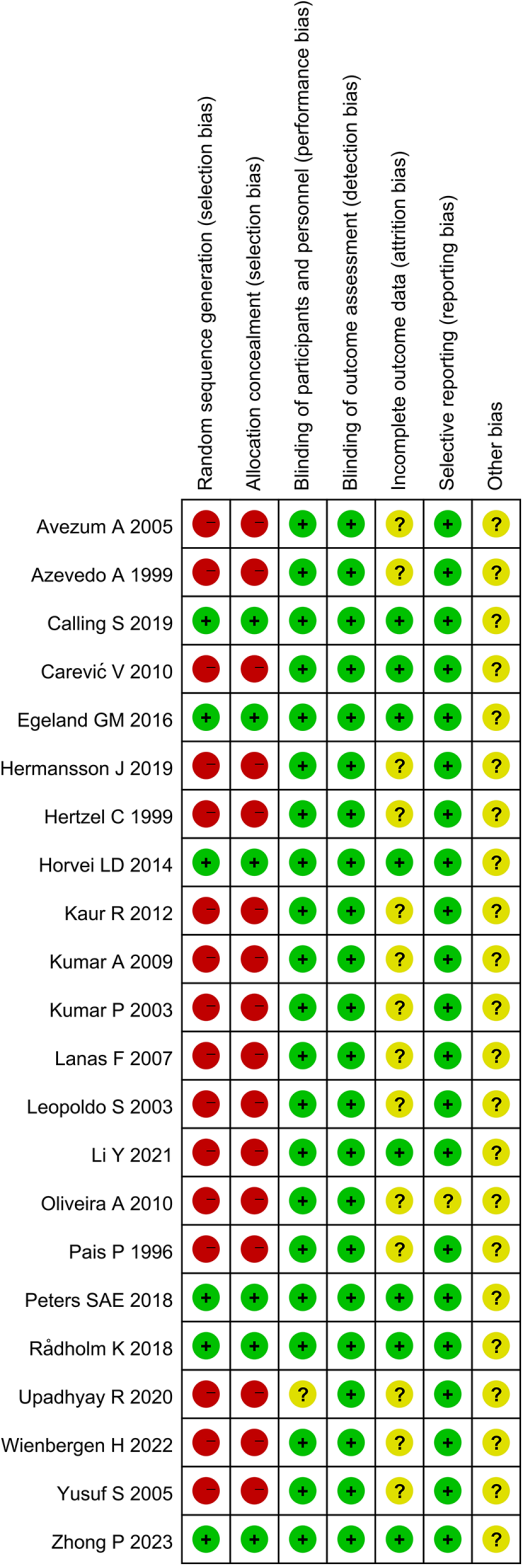
Quality assessment 2. (The green, yellow, and red colors in the fgure represent “low risk,” “unclear,” and “high risk,” respectively.).

To assess the quality of the literature, the NOS was used for scoring, with scores ranging from 7 to 9, indicating good overall quality of the studies. For specific assessment results, please refer to Table 1. Furthermore, Revman 5.3 software was used for further quality assessment of the included studies, examining random sequence generation (selection bias), allocation concealment (selection bias), blinding among participants and personnel (performance bias), blinding in outcome assessment (detection bias), completeness of outcome data (attrition bias), selective reporting (reporting bias), and other bias factors. Given that most of the included studies were case-control studies, the quality assessment was relatively low in terms of random selection and blinding of study subjects, while other aspects including performance bias, detection bias, attrition bias, reporting bias, and other biases were rated as excellent. For specific results, please see Figures 2, 3.

### 3.2 Publication bias

**Figure 4:**
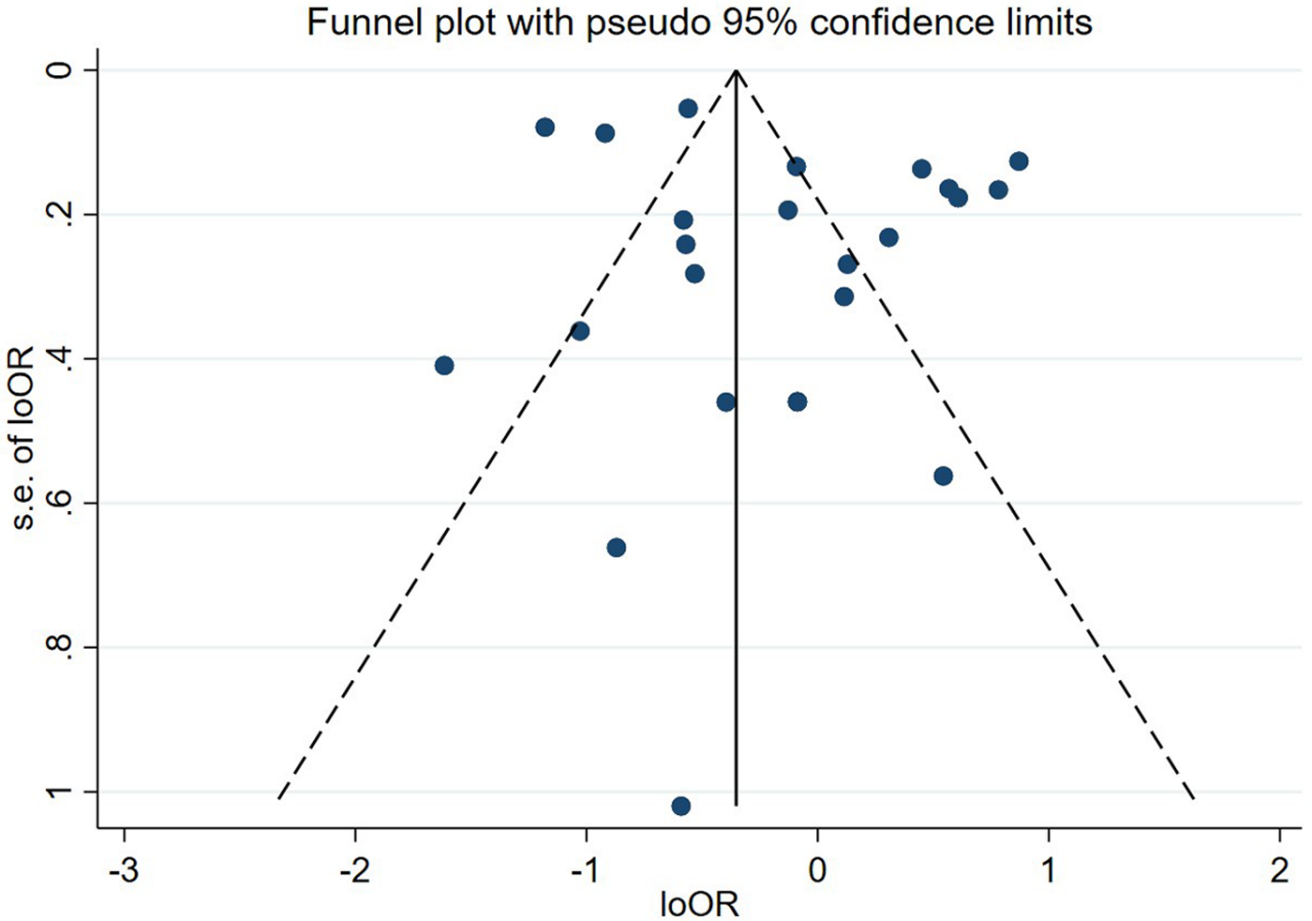
Funnel plot of publication bias among the included studies.

Publication Bias To assess publication bias, we conducted a funnel plot test. The funnel plot (Figure 4) shows that the studies included are relatively symmetrically distributed on the funnel plot, suggesting a lower risk of publication bias in this meta-analysis. However, caution is needed in interpretation, as the assessment of symmetry in funnel plots is somewhat subjective. For details, see Figure 4.

### 3.3 Data analysis results

#### 3.3.1 Relationship between WHR and Mi

**Figure 5:**
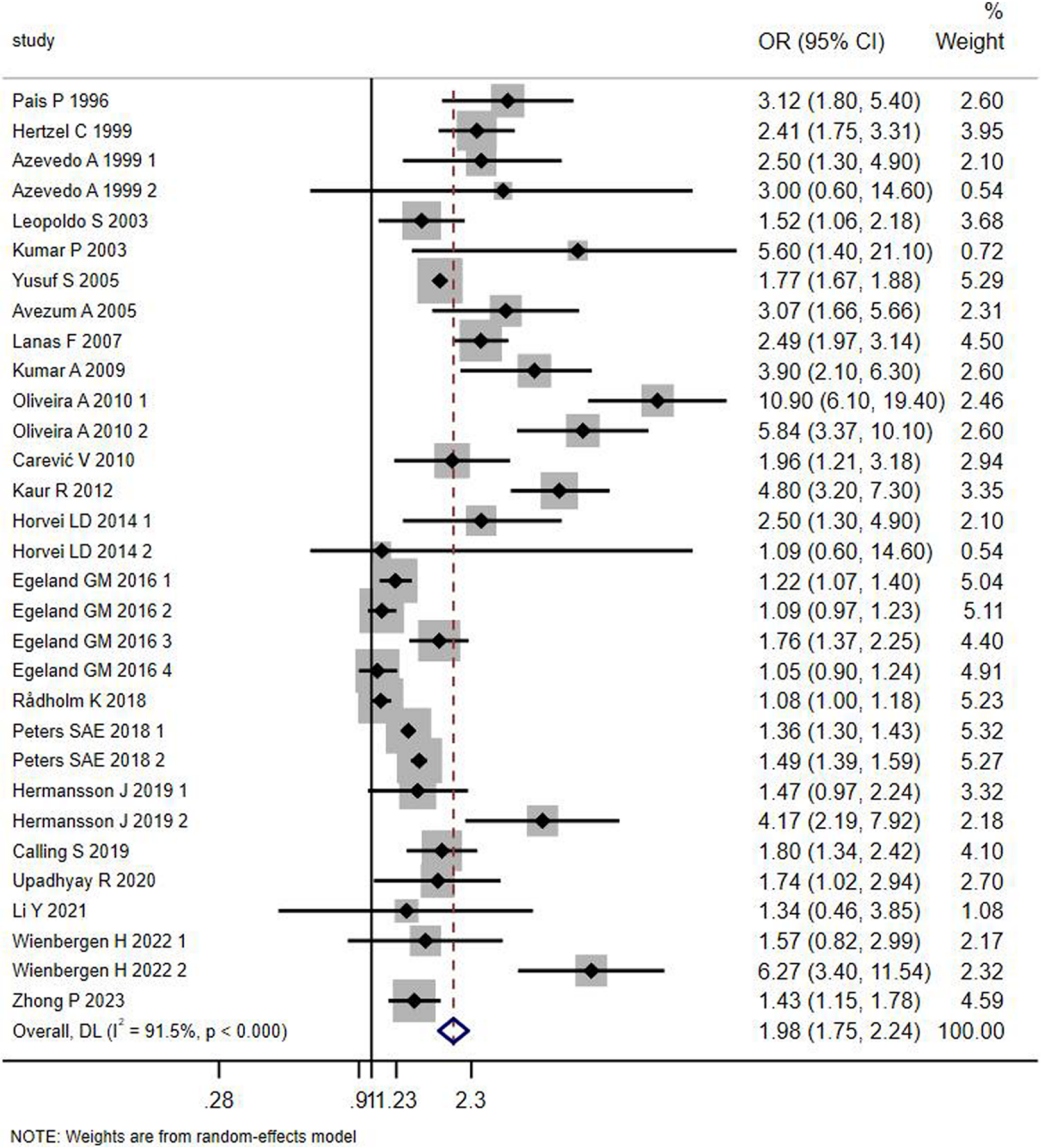
Forest plot of the meta-analysis of the association between waist-to-hip ratio and MI in the general population.

Relationship between WHR and MI The relationship between waist-hip ratio (WHR) and the risk of MI was assessed based on 22 studies of the general population. Considering the heterogeneity of the studies, a random effects model was used for analysis. Some studies were stratified by gender, age, and WHR cut-off values, with numbers 1 to 4 used to differentiate these groups within the same study. The combined results of the random effects model indicated that subjects with a higher WHR are more prone to MI compared to those with a lower WHR. The Cochrane *Q* test showed significant heterogeneity (*P* < 0.0001, *I*^2^ = 91.8%), and the adjusted OR was 1.98 with a 95% CI of 1.75–2.24. Detailed data can be seen in Figure 5. (Note: In the studies by Azevedo A1999, Oliveira A2010, Horvei LD2014, Peters SAE 2018, Hermansson J 2019, “1” represents the male group in the study,“2”represents the female group in the study; In the Wienbergen H 2022 study, “1”represents the group with WHR between 0.87–0.93, “2”represents the group with WHR ≥ 0.93; In Egeland GM2016, “1”represents the male group under 60 years of age,“2”represents the male group over 60 years of age, “3” represents the female group under 60 years of age, “4” represents the female group over 60 years of age.).

#### 3.3.2 Subgroup analyses

##### 3.3.2.1 Gender subgroup analysis

**Figure 6:**
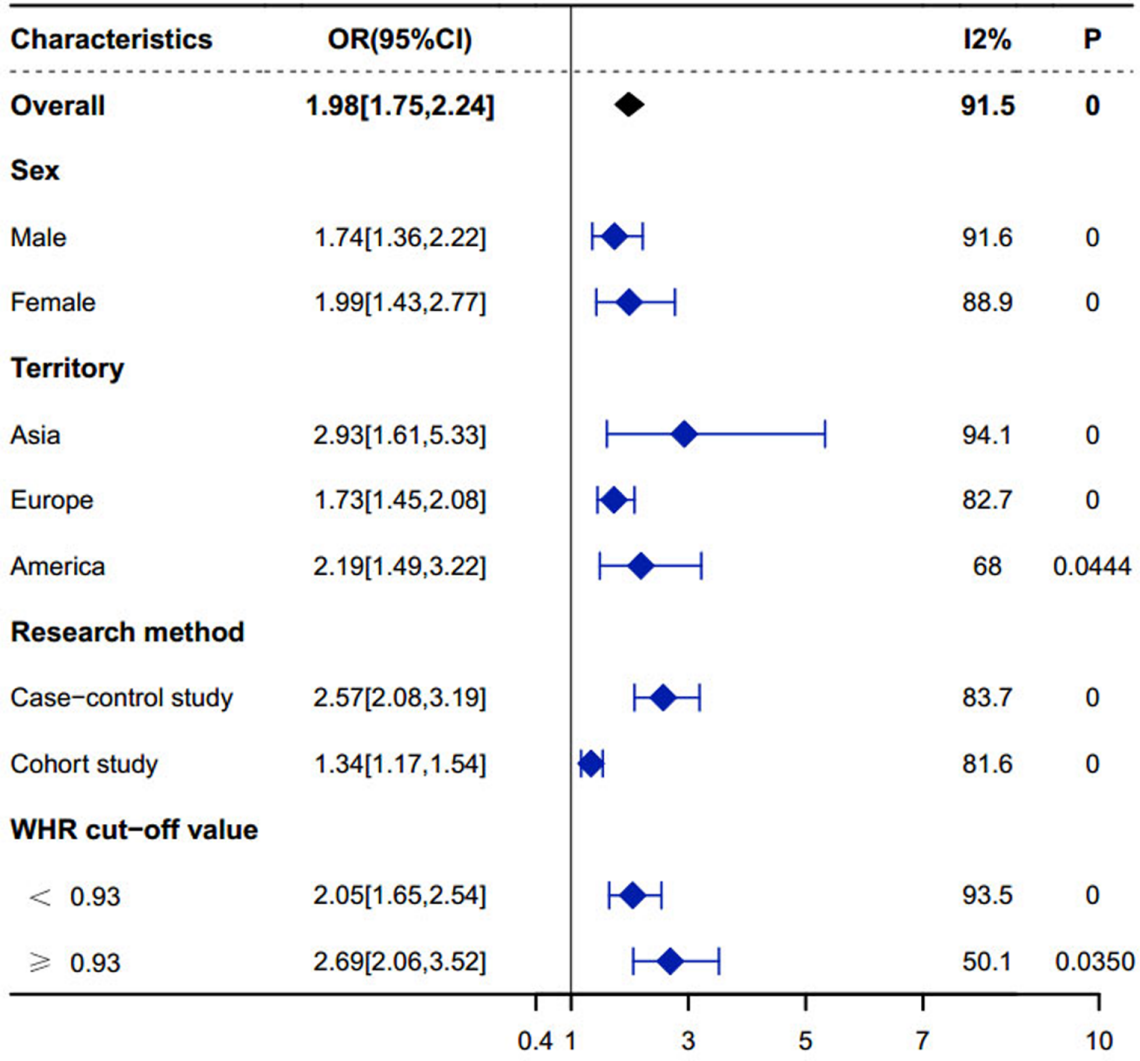
Subgroup analysis.

Subgroup analyses according to gender showed significant associations in both the male (OR: 1.74, 95% CI: 1.36–2.22, *P* < 0.05) and female groups (OR: 1.99, 95% CI: 1.43–2.77, *P* < 0.05), with within-group Cochrane's *Q* test *P*-values were less than 0.05 and *I*^2^ was greater than 50%, suggesting that gender group heterogeneity did not significantly affect outcomes. The combined OR of the female group was greater than that of the male group, indicating that the association between WHR and MI was more significant in females, and thus it can be inferred that WHR is more significant in predicting MI in females. The details are shown in Figure 6. (Note: *P*-value <0.0001 is considered 0).

##### 3.3.2.2 Regional subgroup analyses

According to the results of regional subgroup analysis showed that region 1 = Asia (OR: 2.93, 95% CI: 1.61–5.33, *P* < 0.05), 2 = Europe (OR: 1.73, 95% CI: 1.45–2.08, *P* < 0.05) and 3 = America (OR: 2.19, 95% CI: 1.49–3.22., *P* < 0.05), the Cochrane's *Q*-test *P*-values for within-groups were all less than 0.05, and the *I*^2^ within-groups were all greater than 50%, suggesting that regional subgroup heterogeneity did not have a significant effect on outcomes. As shown in Figure 6. (Note: *P*-value <0.0001 is considered 0).

##### 3.3.2.3 Research methods subgroup analyses

The results of the subgroup analysis according to the research methodology showed that area 1 = case-control study (OR: 2.57, 95% CI: 2.04–3.24, *P* < 0.05), 2 = cohort study (OR: 1.34, 95% CI: 1.17–1.54, *P* < 0.05), and the intragroup Cochrane's *Q*-test *P*-values were all less than 0.05 and *I*^2^ within group was greater than 50%, indicating that heterogeneity in study method grouping did not have a significant effect on outcome. As shown in Figure 6. (Note: *P*-value <0.0001 is considered 0).

##### 3.3.2.4 Subgroup analysis of WHR cut-off value

According to the results of subgroup analysis of WHR critical value showed that region 1 = WHR cut-off value <0.93 (OR: 2.05, 95% CI: 1.65–2.54, *P* < 0.01), 2 = WHR cut-off value ≥0.93 (OR: 2.69, 95% CI: 2.06–3.52, *P* = 0.035), within-group Cochrane's *Q*-test *P*-values were all less than 0.05, and within-group *I*^2^ was greater than 50%, suggesting that heterogeneity of study WHR critical value subgroups did not have a significant effect on outcome. The combined ORs of subgroups with higher WHR critical values were greater than those of subgroups with lower WHR critical values, suggesting that higher WHRs may be more strongly associated with MI. As shown in Figure 6.

#### 3.3.3 Multifactorial meta-regression analysis

To explore the sources of heterogeneity among studies, we further conducted a multifactorial meta-regression analysis. The results of the multifactorial meta-regression analysis showed that the *P*-values for all factors were above 0.05, indicating that factors such as publication year, NOS score, and age did not have a significant impact on the study results. Specific data can be referred to in Table 3. Table 3 shows that the effect size for publication year was −0.18 (*P* = 0.858), with a 95% CI ranging from −0.0551 to 0.0466, indicating that the publication year had no significant effect on the results; the effect size for the NOS score was −1.04 (*P* = 0.321), with a 95% CI ranging from −0.8493 to 0.3022, indicating that the NOS score had no significant effect on the results; the effect size for age was 0.33 (*P* = 0.746), with a 95% CI ranging from −0.0340 to 0.0461, indicating that age had no significant effect on the results. please refer to Table 3.

**Table 3:** Results of the multifactorial meta-regression analysis.

	Effect size	*p*-value	95% confidence interval	
Year of publication	−0.18	0.858	−0.0551273	0.0465668
NOS score	−1.04	0.321	−0.8493396	0.3021809
Year	0.33	0.746	−0.0339729	0.0461412

#### 3.3.4 Sensitivity analysis

**Figure 7:**
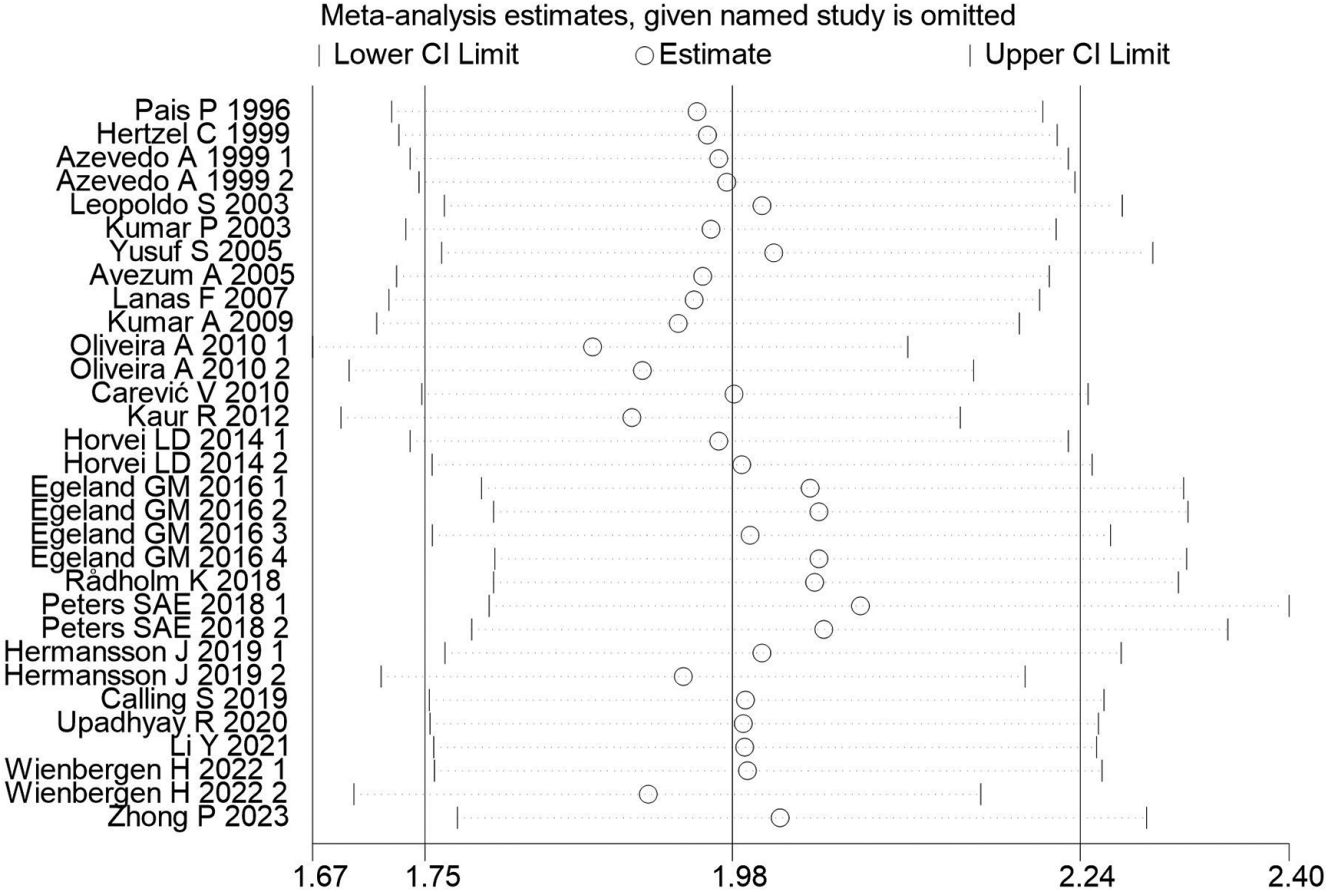
Assessment of meta-analysis stability after exclusion of individual studies. [The horizontal axis represents the exclusion of different studies, and the vertical axis represents the range of changes in the combined effect value (95% CI)].

By sequentially excluding each study and observing the changes in the combined OR value, the results show that the combined OR values are stably distributed between 1.75 and 2.24, indicating that the meta-analysis results are relatively stable. Figure 7 shows that after the exclusion of individual studies, the CI of the combined effect size (OR) did not significantly expand or shift, suggesting that individual studies have limited impact on the overall meta-analysis results. This stability indicates that the meta-analysis results are robust and reliable. For specific results, see Figure 7.

## 4 Discussion

This systematic review and meta-analysis evaluated and summarized the existing evidence on the predictive value of WHR for MI over the past 20 years. We included 22 observational studies from various regions including Europe, Asia, and South America, with a total of 709,093 subjects. The quality of the studies was assessed using the NOS and Revman5.3 software, and the results indicated that the overall quality of the studies was good. The meta-analysis showed that subjects with a higher WHR had a greater likelihood of suffering from MI compared to those with a lower WHR, with an adjusted odds ratio (OR) of 1.98, 95% CI: 1.75–2.24, *I*^2^ = 91.5%, *P* < 0.0001. This suggests that WHR is a promising factor for predicting the risk of MI and has strong predictive power. This indicates that WHR may have significant clinical relevance in identifying high-risk individuals and predicting the burden of MI in the general adult population. Furthermore, these results align with the growing literature (14, 15), supporting WHR as an important indicator in the assessment of MI risk.

In this meta-analysis, by summarizing the existing evidence on the relationship between WHR and MI over the past two decades, we found that WHR has a certain predictive role for the risk of MI. This association may be related to the following mechanisms. Firstly, central obesity refers to a relative abundance of abdominal fat compared to hip fat. A reduction in gluteal region fat is associated with a higher incidence of cardiovascular diseases (38), and abdominal fat is closely related to hypertriglyceridemia and the release of pro-inflammatory cytokines from adipose tissue, implying a higher risk of cardiovascular diseases (39, 40). Secondly, an increase in abdominal fat means an increase in visceral adipose tissue (VAT) (41), which is positively correlated with coronary atherosclerosis (42). Excessive VAT can directly or indirectly cause overactivity of the sympathetic nervous system, as well as abnormal secretion of adiponectin, leptin, and other pro-inflammatory factors, leading to dyslipidemia, a prothrombotic state, insulin resistance, and chronic inflammation, all of which are independent risk factors for cardiovascular diseases (43–47).

To explore the differences in the risk of MI associated with WHR between men and women, we conducted a subgroup analysis by gender. The results showed that an increased WHR is a stronger predictor of MI in women, although the difference is small. This result is consistent with several previous studies (14, 31, 48). In a genome-wide association study of adiposity markers by Peters SAE et al. (31), it was found that visceral fat in women had a stronger correlation with cardiac metabolic risk factors; Ramezankhani A et al. (48) found that WHR increased the risk of cardiovascular events in women more than in men; Qinqin Cao et al (14) found that the OR value of WHR for MI was higher in women in their meta-analysis. Given this result, we should pay more attention to the predictive role of WHR in women for MI, which can help medical institutions and policymakers better tailor prevention and intervention measures for different groups, such as different appropriate cut-off values for WHR in predicting MI in men and women. Researching gender differences can also promote the medical community's attention to women's cardiovascular health issues, improve the diagnosis and treatment level of women's cardiovascular diseases, and optimize the allocation of medical resources and public health policies. However, some studies have reached different conclusions (49). In the study by Hanieh Mohammadi et al. (49)], it was found that the ability of abdominal obesity to be associated with MI was lower in women than in men. Hanieh Mohammadi et al (49) believe this may be related to different fat distributions between genders. Abdominal fat is composed of VAT and subcutaneous adipose tissue (SAT), and generally, men have higher VAT than women, while women have higher SAT. Therefore, compared to women, abdominal obesity is a more direct marker of visceral fat in men, and visceral fat has a stronger correlation with cardiac metabolic risk factors. Therefore, from this mechanism, WHR should have a higher predictive value in men. In summary, no studies have yet clearly explained the mechanisms related to gender differences, so conclusions related to gender need to be interpreted with caution, and further experiments need to be expanded to control more confounding factors, such as age, underlying diseases and medication, lifestyle, hormone levels, etc., to explore gender differences.

To explore whether there are regional differences in the association between WHR and the risk of MI, we conducted subgroup analyses by region. The results suggest that the association between WHR and the risk of MI may be stronger in Asian populations than in American and European populations. This could be related to a combination of factors such as racial differences, local dietary habits, socioeconomic status, medical conditions, lifestyle, and genetic factors (50–52). Particularly, the difference in body fat distribution between regions, with Asian populations tending to accumulate more visceral fat compared to Western populations (53), is closely related to the accumulation of visceral fat, WHR, and the occurrence of cardiovascular diseases. However, the study by Alenaini W et al. (54) found that the differences in visceral fat between populations across states were confounded by differences between rural and urban populations. Therefore, future large-scale prospective cohort studies could be designed to further explore the association and mechanisms between WHR and MI, controlling for confounding factors such as race, living area, body fat distribution, age, and gender.

Given these results, first, the ability of WHR to predict the risk of MI is evident, and medical professionals should consider the key role of WHR in identifying high-risk groups for MI, especially in women. For medical rehabilitation professionals, more attention should be paid to patients' WHR rather than just BMI. Second, in our study, the combined OR value for subgroups with higher WHR cut-off values was greater than for those with lower WHR, suggesting that a higher WHR may be more strongly associated with MI. In the future, the association between different gradients of WHR and the incidence of MI could be further explored to verify whether there is a linear correlation between WHR and the incidence of MI, and to find the WHR cut-off value with the strongest association to establish best practice guidelines. Third, the risks of a high WHR should be explained in patient health education so that patients understand that a high WHR means central obesity, which is closely related to cardiovascular diseases. Normal BMI does not mean the exclusion of the possibility of central obesity, and in daily life, attention should be paid to central obesity indicators such as WHR in addition to weight and BMI. Finally, as an indicator of central obesity, WHR has clinical significance beyond BMI and should receive more attention, especially in the prediction of MI with routine monitoring and early intervention to reduce the risk of MI. Meanwhile, effective treatment optimization needs to be combined with long-term follow-up, which can help minimize the incidence of cardiovascular events (55).

Additionally, this study also assessed the robustness of the meta-analysis results and the risk of publication bias. Sensitivity analysis results (Figure 7) show that after excluding any single study, the combined OR value remains stably distributed between 1.75 and 2.24, suggesting that individual studies have limited impact on the overall meta-analysis results. This stability indicates that the meta-analysis results are highly reliable, further supporting the research conclusion that an increased WHR is significantly associated with an increased risk of MI. At the same time, funnel plot assessment (Figure 6) suggests that the included studies are relatively symmetrically distributed on the funnel plot, indicating a lower risk of publication bias. This somewhat excludes the influence of a positive result publication tendency on the meta-analysis results. However, the assessment of funnel plot symmetry still has a certain subjectivity, so the interpretation of the risk of publication bias should still be cautious.

## 5 Limitations

Firstly, although the combined OR values of subgroups with higher WHR critical values were found to be greater than those of subgroups with with lower WHR critical values in the subgroup analysis, suggesting that a higher WHR may be more strongly correlated with MI, this study only used categorical variable data and could not accurately explain whether there is a linear association between WHR and MI. Secondly, most of the included articles were case-control studies, and there were relatively few cohort studies with high-level evidence, so more cohort studies need to be conducted in the future to increase the credibility of the research results. Thirdly, although various sources of heterogeneity were investigated, no specific cause for heterogeneity was found. The heterogeneity of the studies reduced the reliability of this meta-analysis, and the results should be viewed with caution. Fourthly, different studies used different WHR cutoff values, and it is not possible to determine the effectiveness of a single WHR threshold for predicting MI risk. Fifthly, the sample population was limited. Most of the included studies were case-control studies often focused on a certain hospital or center, and all the studies included were published in English. Therefore, this meta-analysis does not fully represent the entire population, thus limiting the generalizability and extrapolation of the research results. Sixth, the timing of WHR data collection in case-control studies may limit the inference of causal relationships between WHR and MI. This design difference represents a limitation of the current study, potentially affecting the accuracy of event validation. Future research should prioritize prospective cohort designs to enhance the accuracy of causal inference and the assessment of the association between WHR and new incident cases of MI. Seventh, there are differences among researchers in measuring WHR, and it is recommended that future research adopt standardized techniques to objectively measure WHR.

Finally, it should be noted that there was considerable heterogeneity in this meta-analysis. Unfortunately, neither subgroup analysis nor multivariate regression analysis could find the source of heterogeneity. We believe that the reasons for this heterogeneity may include: (1) This meta-analysis combined 22 original studies, which is a large number, and there are differences in the design of each study, participant characteristics, implementation of intervention measures, and outcome measurement standards, leading to inevitable heterogeneity; (2) The heterogeneity may be related to the type of MI, differences in the standards for diagnosing MI between studies, inconsistencies in the measurement methods of WHR and covariates, and Comorbidities and and follow-up measures and different follow-up times, but since the original studies did not provide corresponding data, subgroup analysis could not be conducted; (3) Potential differences in event definitions and collection methods among different studies may lead to bias. Although heterogeneity reduces the reliability of the meta-analysis, the sensitivity analysis showed that by excluding studies one by one, regardless of which study was excluded, the combined results were stable on both sides of the median line. There was no reversal of results, and the meta-analysis therefore has stability. In summary, although there is heterogeneity in this study, it still has reference value. We should treat these results with caution, and more prospective cohort studies can be set up in the future to further verify this conclusion.

## 6 Conclusion

WHR is an important predictor of MI risk. Individuals with a high WHR have a signifcantly higher risk of MI than those with a low WHR, an association that is more signifcant in women.Furthermore, the higher the WHR critical value, the stronger the association with MI, suggesting apossible dose-response relationship.

Clinical medical staff should therefore pay attention to the measurement and monitoring of WHR, and use it as an important means of assessing and preventing MI risk, especially for women and individuals with a signifcantly increased WHR. Morehigh-quality prospective studies are needed to further verify thepredictive value of WHR and optimize its application in MI risk assessment.

Future research should combine WHR with other risk factors to better guide the prevention and management of MI.

## Data Availability

The original contributions presented in the study are included in the article/supplementary material, further inquiries can be directed to the corresponding author/s.
